# Mothers and Nurses

**Published:** 1875-06

**Authors:** 


MOTHERS AND NURSES should labor to acquire an habitual composure, self-possession, and presence of mind, and, as far as possible, to impart the same to children or invalids; to be always quiet; quick in applying the necessary remedies; not yielding to sudden alarms and agitations; never indulging in the injurious habit of screaming or uttering exclamations on the various accidents of a nursery or sick-room, nor urging as a plea for such failures *a weakness of nerves.* This, in the present day, is often brought forward as a cover for infirmities which are rather to be condemned and resolutely overcome, than palliated or indulged.

